# A Trial of the Efficacy, Safety and Impact on Drug Resistance of Four Drug Regimens for Seasonal Intermittent Preventive Treatment for Malaria in Senegalese Children

**DOI:** 10.1371/journal.pone.0001471

**Published:** 2008-01-23

**Authors:** Cheikh Sokhna, Badara Cissé, El Hadj Bâ, Paul Milligan, Rachel Hallett, Colin Sutherland, Oumar Gaye, Denis Boulanger, Kirsten Simondon, François Simondon, Geoffrey Targett, Jo Lines, Brian Greenwood, Jean-François Trape

**Affiliations:** 1 Institut de Recherche pour le Développement, Dakar, Senegal; 2 Université Cheikh Anta Diop de Dakar, Dakar, Senegal; 3 London School of Hygiene & Tropical Medicine, London, United Kingdom; Royal Melbourne Hospital, Australia

## Abstract

**Summary:**

In the Sahel, most malaria deaths occur among children 1–4 years old during a short transmission season. A trial of seasonal intermittent preventive treatment (IPT) with sulfadoxine-pyrimethamine (SP) and a single dose of artesunate (AS) showed an 86% reduction in the incidence of malaria in Senegal but this may not be the optimum regimen. We compared this regimen with three alternatives.

**Methods:**

2102 children aged 6–59 months received either one dose of SP plus one dose of AS (SP+1AS) (the previous regimen), one dose of SP plus 3 daily doses of AS (SP+3AS), one dose of SP plus three daily doses of amodiaquine (AQ) (SP+3AQ) or 3 daily doses of AQ and AS (3AQ+3AS). Treatments were given once a month on three occasions during the malaria transmission season. The primary end point was incidence of clinical malaria. Secondary end-points were incidence of adverse events, mean haemoglobin concentration and prevalence of parasites carrying markers of resistance to SP.

**Findings:**

The incidence of malaria, and the prevalence of parasitaemia at the end of the transmission season, were lowest in the group that received SP+3AQ: 10% of children in the group that received SP+1AS had malaria, compared to 9% in the SP+3AS group (hazard ratio HR 0.90, 95%CI 0.60, 1.36); 11% in the 3AQ+3AS group, HR 1.1 (0.76–1.7); and 5% in the SP+3AQ group, HR 0.50 (0.30–0.81). Mutations associated with resistance to SP were present in almost all parasites detected at the end of the transmission season, but the prevalence of *Plasmodium falciparum* was very low in the SP+3AQ group.

**Conclusions:**

Monthly treatment with SP+3AQ is a highly effective regimen for seasonal IPT. Choice of this regimen would minimise the spread of drug resistance and allow artemisinins to be reserved for the treatment of acute clinical malaria.

**Trial Registration:**

Clinicaltrials.gov NCT00132548

## Introduction

In Sub-Saharan Africa, malaria remains the most common cause of morbidity and mortality and is still estimated to cause one million deaths a year, primarily in young children [1]. Insecticide-treated bednets (ITNs) can reduce mortality and morbidity from malaria substantially [2] but they are only partially effective and achieving high levels of coverage with ITNs has proved difficult. Other preventive strategies are needed.

Administration of antimalarial chemoprophylaxis to the whole paediatric population can reduce malaria morbidity and all-causes mortality substantially [3] but this approach to malaria control has not been adopted widely because of concerns over the enhancement of drug resistance, impairment of naturally acquired immunity and difficulties in implementation. Intermittent preventive treatment (IPT), the administration of an antimalarial drug or drug combination in curative doses at specific time points (for example antenatal clinic visits or visits for routine immunisation) offers a potential way of achieving some of the gains provided by chemoprophylaxis whilst limiting some of its potential drawbacks [4]. Drugs used for IPT must be very safe as well as effective. Several studies have shown that IPT given with routine immunisation during the first year of life is effective in reducing the incidence of clinical malaria and of anaemia [5]–[9]. In Tanzania, approximately 50% protection against clinical attacks of malaria and anaemia was achieved during the first year of life using sulfadoxine-pyrimethamine (SP) and this protection was sustained during the following year in the absence of any further drug administration [5], [6]. A second study undertaken in Tanzania in which amodiaquine (AQ) was given routinely at growth monitoring visits achieved similar results [7].

However, more recent studies of IPT with SP conducted in Ghana and Mozambique have given less marked reductions in the incidence of malaria or anaemia and no persistence of protection beyond the period of drug administration [8], [9]. Further trials of IPT in infants using alternative drugs to SP are under way in Kenya and Tanzania under the auspices of the IPTi consortium [10]. Sufficient data should have been collected within the next year to determine whether IPT in infants is sufficiently effective to warrant introduction into the routine expanded programme of immunisation (EPI).

In many parts of Africa, especially those where transmission is seasonal, the main burden of malaria is not in infants but in older children [11], [12] and IPT in infants would not be expected to have a major impact on the overall burden of malaria in children, unless protection persists beyond the period of drug administration as observed in Tanzania [6]. Thus, we and others have explored the feasibility of using the IPT approach to prevent malaria in older children [13], [14]. In Mali, administration of two doses of SP to children under five years of age during the malaria season resulted in a 40% reduction in clinical attacks of malaria [13]. In 2002, children aged 3–59 months resident in Niakhar, a typical Sudano-Sahelian area of rural Senegal where malaria transmission is seasonal, were given a single dose of SP plus one dose of artesunate on three occasions at monthly intervals during the peak malaria transmission season. This resulted in an 86% reduction in clinical attacks of malaria in children without any rebound in the incidence of malaria during the following year [14]. At the end of the malaria transmission season, the proportion of malaria parasites carrying molecular markers for resistance to pyrimethamine and sulfadoxine was increased in children who had received IPT but the number of children carrying resistant parasites was less than in the control group because fewer children had parasitaemia. This increase was seen despite the fact that children had received an artemisinin-based combination therapy (ACT). Because of these findings we have investigated whether other drug regimens might be equally effective in preventing malaria but more effective in preventing the emergence of resistant parasites. The regimens were chosen for evaluation for the following reasons. A combination of two drugs, as opposed to monotherapy, was used to reduce the risk of the emergence of resistant parasites. Amodiaquine was chosen as a component of two of the combinations as this drug is still effective in Senegal and is moderately long lasting. Treatment trials have shown that SP combined with three daily doses of amodiaquine is more effective at preventing 28-day post-treatment relapses or recrudescence than SP combined with artesunate [15] and an important objective for this trial was to determine whether this would also be the case for IPT. A one-day regimen of SP plus artesunate was included as a comparator as this was the schedule used in a previous IPT trial in Senegal.

## Materials and Methods

### Study area

The study was carried out in Niakhar, a rural district in central Senegal with 33,000 inhabitants that has for several decades been a regional observatory for population and health studies [16]. In this area, rains are concentrated over a three-month period from July to the beginning of October. Malaria transmission, almost exclusively by *Anopheles arabiensis (A. gambiae s.l.)*, is strictly seasonal and concentrated in September and October. The parasite rate in children is usually less than 50% for most of the year but can reach figures as high as 80% at the end of the rainy season [17]. *P. falciparum* is the dominant species. Eighty per cent of malaria deaths occur among children 0–4 years of age. From 1988 to 1991, the average malaria mortality rate was 5.4 per thousand per year among children between 0 and 4 years of age. The emergence of chloroquine resistance in 1992 [18] was associated with a dramatic increase in malaria mortality, which averaged 12.4 per thousand per year among children under 5 years of age during the period 1992–1995 [19].

### Study design

An open label trial was conducted which compared four treatment regimens: one dose of SP plus one dose of AS (SP+1AS); one dose of SP plus AS given for three days (SP+3AS); one dose of SP plus AQ given for three days (SP+3AQ); AQ plus AS both given for three days (3AQ+3AS). These regimens were chosen for evaluation because resistance of *P. falciparum* to SP and AQ is known to be only modest in the study region and because the use of combination therapy should reduce the risk of the emergence of resistant parasites. Eligible participants were allocated to receive one of the study combinations in early September, early October and early November 2004.

### Enrolment, treatment allocation and drug distribution

Community meetings were held in the study area to explain the aims and procedures of the study. Information about the trial was provided in local languages (Serer and Wolof). Oral consent was sought from a parent or guardian. Fourteen villages were chosen for logistic convenience and a list of 2102 children living in these villages and who would be aged between 6 and 59 months at the time of the first treatment in September was obtained from the Niakhar demographic surveillance database. These children were invited to be screened for participation in the trial between July and August 2004. 5 refused to participate, leaving 2097 children who had parental consent to participate and were enrolled. They were systematically allocated in the order in which they had been screened in sequence in blocks of four to receive SP+1AS, SP+3AS, 3AQ3+3AS, or SP+3AQ. The children were issued with an ID card to facilitate identification at subsequent contacts at home or in a health centre. When eligibility was checked again on the day of first treatment in September, 68 children had left the study area, 6 had died, and 3 were excluded because of illness (2 with acute respiratory illness and one with chronic illness), leaving 2020 children in the study. None had a history of allergy to the study drugs.

Study drugs for the three treatment course were placed in envelopes bearing the child's ID number. Dosing was according to weight in two weight groups, <12 kg and > = 12 kg. The dosage of SP (tablets of 500 mg sulfadoxine and 25 mg pyrimethamine) was ½ tablet (<12 kg) or 1 tablet (> = 12 kg), dosage of AS (50 mg tablets) was 1 tablet per dose (<12 kg) or 1.25 tablets (> = 12 kg), and dosage of AQ (200 mg tablets), ¾ tablet for children <12 kg and 1.25 tablets for children > = 12 kg. Treatment allocation, packaging, and drug administration were done by staff that played no part in evaluation of study subjects. The trial was not blinded; as amodiaquine tablets were yellow parents of children in this group would have been aware of their treatment allocation; but field staff involved in evaluation of study subjects, and laboratory staff, were unaware of a child's treatment group.

Children were given the first dose of treatment at the health centre. Subsequent doses were given at home by study staff. For young children, tablets were crushed and mixed with water prior to administration. Participants were observed for 15 minutes after drug administration; if the child vomited the dose was repeated. Before administration of the first dose, children were examined and any child who had malaria, defined as fever (temperature by auricular thermometer > = 38°C) or a history of fever or vomiting within the last 24 hours together with parasitaemia at any density was not given the IPT treatment that month but was treated with artemether/lumefantrine (Co-Artem®).

### Patient follow-up, assessment procedures and clinical management

Participants were visited at home each week over three months from September to December to record episodes of malaria. At each visit, axillary temperature was measured and a blood film made if the child was febrile (> = 38°C), or had a history of fever or vomiting in the previous 24 hours [20]. Children who were slide positive were treated with Coartem over three days. To ensure detection and prompt treatment of malaria at other times, parents were encouraged to bring their child to the health centre if the child was ill. Each parent was given a consultation ticket which entitled their child to free consultation at the nearest health centre where physical and parasitological examinations were done promptly. Children who were treated for malaria were revisited at least once a day until the third day after treatment to monitor the child's recovery. An experienced supervisor checked the performance of study field-workers by re-visiting in the afternoon selected participants who had been seen in the morning, to check for malaria symptoms and the accuracy of data recording.

At the time of the first drug administration (September 2004) and one month after the third and last drug administration (December 2004), a finger-prick blood sample was taken from each child for measurement of haemoglobin concentration, detection of parasitaemia and for parasite genotyping. Children who were anaemic (Hb<9 g/dL) at the September survey were given iron supplementation (children with Hb<9 g/dL were given one tablet of iron per day for four weeks, children with Hb<7 g/dL were given, in addition, multivitamins and mebendazole for 3 days, according to national guidelines).

### Laboratory methods

At the time of the cross-sectional surveys in September and December, filter paper blood samples were collected and stored for analysis of molecular markers for resistance. Parasite densities were estimated in thick blood films, assuming an average white blood cell count of 8000 per µl. All slides were double read; for acute cases, to permit prompt treatment, treatment was decided on the basis of the first reading. Haemoglobin (Hb) concentration was measured in a HemoCue Machine®. DNA was extracted from filter papers obtained from parasite positive children using the chelex technique [21]. Mutations in the *pfdhfr* and the *pfdhps* genes were identified using sequence specific oligo-nucleotide probing (SSOP) following a method adapted from that of Pearce et al. [22].

### Assessments of adverse events

Adverse events were defined as any signs or symptoms which first appeared, or increased in severity, within seven days of drug administration. Any serious adverse events such as a death or admission to hospital was thoroughly investigated and reported within 24 hours to the Data Safety and Monitoring Board (DSMB) established to support the trial. To determine the incidence of minor adverse events following drug administration all children were visited at home seven days after each round of drug administration and an adverse events questionnaire completed. The medical team followed up participants with adverse experiences until the event was cured or had stabilised. Parents were also strongly advised to inform project field staff or to go to the nearest health centre if their child became ill following drug administration.

### Statistical analysis

The primary study endpoint was the incidence of the first or only episode of malaria, defined as a parasitaemia of 3000/µL or more together with fever (temperature measured by auricular thermometer ≥38.0°C) or a history of fever or vomiting in the previous 24 hours. This definition has been shown in nearby Ndiop to have optimum sensitivity and specificity for diagnosis of clinical malaria in children under active surveillance (Trape JF and C Rogier, unpublished). Secondary endpoints were the incidence of adverse events, the mean Hb concentration, the prevalence of asexual parasitaemia, and the prevalence of *P.falciparum* genotypes associated with resistance to sulfadoxine and to pyrimethamine, recorded in the cross-sectional survey in December.

Sample size was calculated for comparison of each treatment group to the group who received SP+1AS, the regimen used in the trial conducted previously in

Niakhar [14], firstly to establish non-inferiority with respect to malaria incidence (we assumed an expected cumulative incidence of malaria of 7% in all groups). We chose a non-inferiority margin of 5%, and determined the sample size for 90% power and 95% confidence, allowing for 10% loss to follow-up. Secondly, the study was designed to have adequate power to detect a reduction in the overall prevalence of drug-resistant parasite genotypes, assumed to be about 13% (based on a parasite prevalence of 14% in the SP+1AS group, 95% of which would be carrying the triple *dhfr* mutation associated with resistance to pyrimethamine). Five hundred and twenty subjects were required for each group.

The primary analysis includes all individuals who were allocated to treatment provided that they received at least one follow-up visit, regardless of the number of treatments received. Data have been analysed according to the treatment group to which a child was allocated (intention to treat). The analysis plan, finalised and approved by the DSMB before the treatment code was broken, specified that the primary analysis should include adjustment for the covariates age and bednet use. When the data were reviewed, geographical area was found to be strongly associated with the risk of malaria and was included as a covariate. For analysis, villages were grouped into two areas, one with a low and one with a high malaria risk. To determine non-inferiority within the specified 5% margin, each treatment group was compared to the SP+1AS group using the 95% confidence interval for the difference in malaria risk. When the 95% interval excludes −5% but also lies above zero, a 2-sided P-value for the test of superiority was calculated. In addition the hazard ratio was estimated using Cox's regression, with observations for children who were lost to follow up (or died) being censored on the date they were last seen (or the date of death). In the primary analysis, only cases with parasitaemia ≥3000/µl were included; this is a more specific case definition but the analysis does not take account of the effects of drug treatment in malaria cases with densities below this cut-off. Therefore in a secondary analysis, all treated cases (with parasitaemia at any density) were included. Differences in mean Hb concentrations between treatment groups in December were estimated using analysis of covariance, adjusting for the Hb measurement at enrolment. Incidence of adverse events in each treatment group was compared using the chi-squared test. All analyses were done with STATA software version 8 (College Station, TX, USA).

### Ethics

In view of the high level of protection against malaria obtained in a previous trial in the study area using SP+one dose of AS it was not considered justifiable to include a placebo group in the study. The study was approved by the ethics review boards of the Senegalese Ministry of Health and the London School of Hygiene & Tropical Medicine. The trial was monitored by a DSMB and an independent trial monitor.

The protocol for this trial and supporting CONSORT checklist are available as supporting information; see Checklist S1 and Protocol S1.

## Results

**Figure 1:**
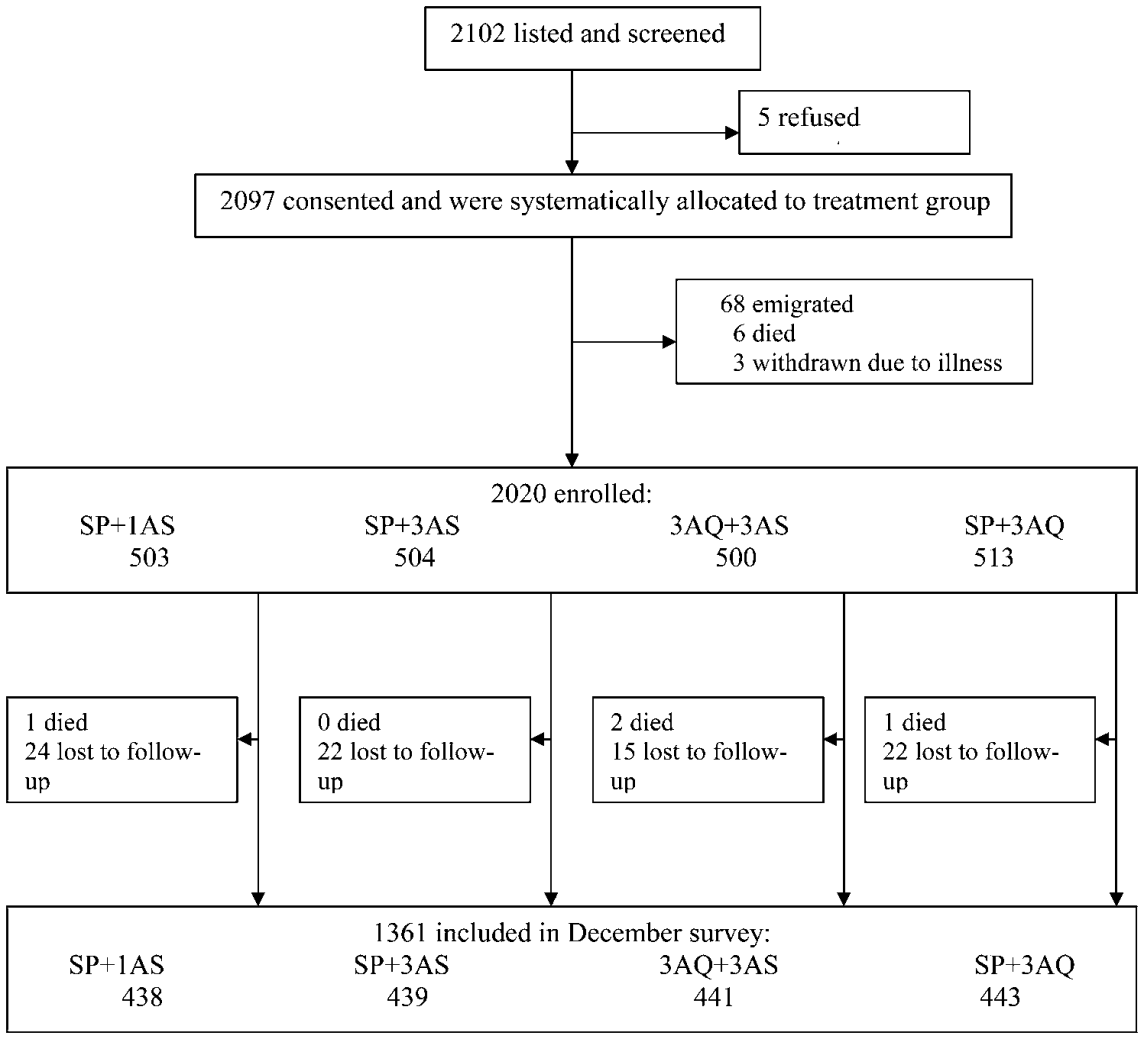
Trial profile.

Two thousand one hundred and two children aged 6–59 months were screened and allocated to receive monthly IPT with SP+1AS, SP+3AS, 3AQ+3AS or SP+3AQ. Two thousand and twenty children (96%) who were enrolled, treated and followed up for at least one visit were included in the primary analysis. Surveillance began on September 6^th^ 2004, the first IPT treatments were given in the period 6^th^–16^th^ September 2004, and follow-up ended on November 28^th^ 2004. Of these 2020 children, four children died and a further 83 were lost to follow up before the end of the surveillance period (Figure 1). Among the 2020 included in the analysis, 1 child in the SP+1AS group received 2 treatments of SP+3AQ and missed their third treatment; 18 children (8 in the SP+1ASgroup, 4 in the SP+3AS group, 4 in the 3AQ+3AS group and 2 in the SP+3AQgroup) received the wrong treatment on one or more occasions. Twenty-nine children received no IPT doses and 1503 (74%) received all 3 treatments. Vomitting of the study medication was more common in the groups receiving amodiaquine. The percentage of children that vomitted the first dose of the treatment round was 0.73% in the SP+1AS group (10 occasions out of 1362), 0.74% (10/1353) in the SP+3AS group,2.3% (31/1357) in the AQ/AS group and 4.1% (58/1414) in the SP+3AQ group.

On entry into the trial, the four study groups were well matched for variables such as age, residence, bednet use and prevalence of *P. falciparum* asexual stage parasitaemia (Table 1). At enrolment, 45% of children (883/1974 tested) had a Hb concentration<9 g/dl and received iron supplementation; the proportion who received supplementation was similar in each treatment group (Table 1). Use of bednets was limited, 18% (359/1968) slept under an intact or impregnated bednet.

**Table 1:** Characteristics of the study subjects at enrolment by treatment group.

	SP+1AS	SP+3AS	3AQ+3AS	SP+3AQ
N	503	504	500	513
Male (%)	47%	49%	49%	49%
Mean (range) age (months)	32 (5,60)	32 (6, 60)	32 (6, 60)	34 (6, 60)
Age				
<12 months	50 (10%)	47 (9%)	58 (12%)	52 (10%)
12–23 months	130 (26%)	116 (23%)	109 (22%)	116 (23%)
24–35 months	120 (24%)	134 (27%)	109 (22%)	112 (22%)
36–47 months	106 (21%)	113 (22%)	123 (25%)	120 (23%)
48–59 months	97 (19%)	94 (19%)	101 (20%)	113 (22%)
Area				
High EIR	152 (30%)	153 (30%)	151 (30%)	152 (30%)
Low EIR	351 (70%)	351 (70%)	349 (70%)	361 (70%)
Bednet use†	17% (85/490)	21% (104/503)	17% (85/489)	17% (85/486)
Hb concentration in September (mean g/dl, range)	9.1 (3.9–13.7)	9.0 (3.4–14.0)	9.1 (4.7–13.4)	9.1 (4.2–12.8)
% Hb<9 g/dl in September	46% (230/497)	45% (220/486)	44% (218/492)	43% (215/499)
Asexual *P.falciparum* prevalence in September	28% (129/459)	27% (123/448)	28% (129/457)	26% (120/466)
Percent of tested samples in September positive for dhfr triple mutation (51 59 and 108)	79% (66/85)	72% (53/74)	75% (65/86)	86% (68/79)
Percent of tested samples in September positive for dhps mutation (437) (none with 540)	52% (51/98)	50% (50/81)	49% (42/85)	56% (40/71)
Gametocyte prevalence in September	7.2% (33/459)	7.1% (32/448)	5.9% (27/457)	7.5% (35/466)

† sleeps under an intact or impregnated net.

EIR = entomological inoculation rate

### Malaria incidence

**Figure 2:**
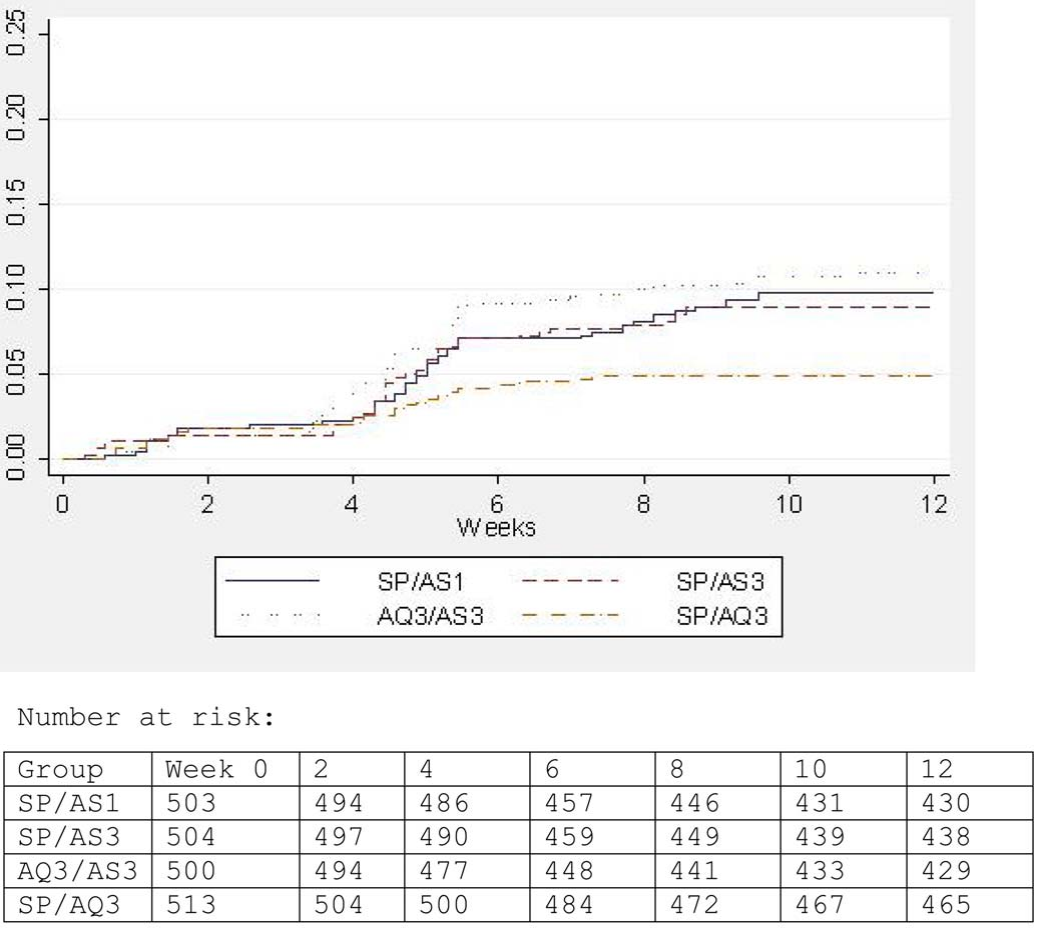
Kaplan Meier estimate of the cumulative incidence of malaria during the 3 months of follow-up.

The overall incidence of clinical attacks of malaria in study children was 9.1% (171/2020). Three children had two malaria episodes separated by a period of more than 28 days, two children in the 3AQ+3AS group and one child in the SP+3AS group. No child had more than two episodes. In addition, there were 7 children who had second episodes within 28 days of the primary malaria attack (two in the SP+1AS group, two in the SP+3AS group, two in the 3AQ+3AS group, and one in the SP+3AQ group) . Incidence by treatment group is shown in Table 2. The distribution of malaria episodes over time is shown in Figure 2.

**Table 2:** Incidence of malaria by age, bednet usage, area of residence and treatment group.

	Cumulative incidence*	Crude Hazard Ratio#	Adjusted Hazard Ratio (95%CI)
Treatment group			
SP+1AS	48/503 (9.5%)	1	1
SP+3AS	44/504 (8.7%)	0.93	0.90 (0.60–1.36)
3AQ+3AS	54/500 (10.8%)	1.15	1.13 (0.76–1.67)
SP+3AQ	25/513 (4.9%)	0.51	0.50 (0.31–0.81)
Age at enrolment			
<12 months	10/207 (5.0%)	1	1
1–2 yrs	24/471 (5.2%)	1.2	1.2 (0.56–2.6)
2–3 yrs	49/475 (10.5%)	2.3	2.4 (1.2–4.9)
3–4 yrs	48/462 (10.5%)	2.3	2.4 (1.2–4.9)
4–5 yrs	40/405 (10.0%)	2.3	2.4 (1.2–5.0)
Bednet use †			
Yes	20/359 (5.6%)	1	1
No	148/1609 (9.3%)	1.7	1.6 (1.0–2.6)
Area			
Low EIR	26/608 (4.4%)	1	1
High EIR	145/1412 (10.4%)	2.5	2.6 (1.7–4.0)

* risk differences compared to the SP+1AS group are: SP+3AS, 0.81% (95%CI −2.7%, +4.4%); 3AQ+3AS −1.3% (−5.0%, +2.5%); and SP+3AQ 4.7% (+1.5%, +7.8%).

# based on data for 1968 individuals who were followed up and had covariate data. Crude hazard ratios, including those with missing covariate data, were 0.92, 1.14 and 0.50 for SP+3AS, 3AQ+3AS and SP+3AQ respectively.

† defined as sleeping under an intact or impregnated net.

Cumulative incidence of malaria was similar in the three treatment groups that included artesunate in both the intention to treat and per protocol analyses (i.e. analysis restricted to include only children who received three treatments; data not shown). The incidence of malaria in the SP+3AQ group (4.9%) was lower than in the group that received SP+1AS, with a 95% confidence interval for the risk difference that excludes zero. The hazard ratio was 0.50, (95%CI 0.30–0.81), P = 0.006. Malaria incidence was lower in children under 2 yrs than in children 2–5 yrs of age, and incidence was reduced in children who slept under a bednet and in children who lived in areas where the entomological inoculation rate (EIR) was known to be low. Hazard ratios for treatment differences adjusted for these effects were similar to unadjusted estimates (Table 2). There was no association between iron supplementation and malaria incidence, and there was no evidence of interaction between treatment group and bednet use, treatment group and EIR, and treatment group and whether the child received iron supplementation at enrolment (data not shown). In a secondary analysis of all malaria episodes (with parasites at any density), similar results were obtained (data not shown).

### Anaemia

Mean Hb concentration at the cross-sectional survey in December was higher than in September in each of the treatment groups. The rise was most marked in the children who were anaemic (Hb<9 g/dL) in September and received iron supplementation. The Hb concentration in December was slightly greater in the 3AQ+3AS and SP+3AQ groups (10.0 g/DL in each group) than in the SP+1AS group (9.7 g/dL) (difference, adjusted for September values, 0.33 g/dL [95%CI 0.15 to 0.50]). Three children were severely anaemic (Hb<5 g/dL) at the time of the December survey, one in the SP+1AS group and two in the SP+3AS group.

### Impact of intermittent treatment on the prevalence of malaria parasitaemia and on markers of resistance to SP

At the end of the malaria transmission season, the prevalence of asexual stage *P.falciparum* parasitaemia was low in all groups but was higher in the SP+1AS group (8.7%) than in the other groups and was lowest in the SP+3AQ group (1.1%) (Table 3). The prevalence of *P. falciparum* gametocytaemia was less than 1% in all groups. *Pfdhfr* mutations at positions 51, 59, 108 and a *Pfdhps* mutation at position 437 were detected in almost all parasite-positive samples at the time of the December survey (Table 3).

**Table 3:** The prevalence of parasitaemia, and proportion of parasitaemic children carrying parasites positive for DHFR and DHPS mutations associated with resistance to pyrimethamnine and suphadoxine in December, after administration of IPT during the preceding 3 months.

	Asexual parasite prevalence	Risk difference (95%CI)	Gametocyte prevalence	DHFR triple mutation	DHPS 437
SP+1AS	8.7% (38/438)	Reference	4/438	24/24	27/28
SP+3AS	1.8% (8/439)	6.9% (3.9%,9.8%)	0/439	4/5	3/3
3AS+3AQ	3.4% (15/441)	5.3% (2.1%, 8.4%)	1/441	9/9	6/9
SP+3AQ	1.1% (5/443)	7.5% (4.7%, 10.4%)	4/443	5/5	5/5

### Adverse events

The incidence of adverse reactions was recorded during the three days after each treatment round (Table 4). Adverse events were more common in the SP+3AQ and 3AQ+3AS groups than in the SP+1AS and SP+3AS groups, with fever and vomiting the most commonly reported adverse events. No severe skin or neurological reactions were reported. A rash was noted in 8 children; in none was this suggestive of Stevens Johnson syndrome. Two children were treated for severe malnutrition. Only four children died during the period of surveillance. An 18-month old boy in the SP+1AS group died of burns, a 2 year old girl in the 3AQ+3AS group died of dysentery and a 12 month old girl in this group died of a febrile illness characterized by vomiting and diarrhoea. She was treated with both an antimalarial and an antibiotic but died 4 days after the start of treatment. The final death was that of a 5-year old boy in the SP+3AQ who died of a febrile illness and convulsions; his blood film was negative. The mortality rate of 2 per 1000 (4/2020) among the study children was lower than the rate of 6 per 1000 in the same age group in the rest of the population in the demographic surveillance area during the same period (A Marra, personal communication).

**Table 4:** Adverse events recorded during the week after each treatment by treatment group.

	SP+1AS	SP+3AS	3AQ+3AS	SP+3AQ	P-value*
	N = 504	N = 501	N = 495	N = 509	
% children (n) with any symptom (95% CI)	8.1% (41) (6.9%, 10.9%)	9.6% (48) (7.1%, 12.5%)	24% (120) (21%, 28%)	32% (164) (28%, 36%)	<0.001
convulsion	0% (0)	0.2% (1)	0% (0)	0% (0)	0.390
agitation	0.6% (3)	0.4% (2)	2.6% (13)	5.7% (29)	<0.001
headache	1.2% (6)	0.4% (2)	2.4% (12)	5.7% (29)	<0.001
fever	5.0% (25)	4.2% (21)	9.9% (49)	11.2% (57)	<0.001
vomited	1.6% (8)	1.8% (9)	6.3% (31)	11.2% (57)	<0.001
rash	0.2% (1)	0.4% (2)	0.2% (1)	0.8% (4)	0.405
dizziness	0% (0)	0% (0)	0.4% (2)	0.8% (4)	0.064
diarrhoea	2.6% (13)	2.8% (14)	5.3% (26)	4.7% (24)	0.060
Abdominal pain	0.2% (1)	0% (0)	0.8% (4)	2.0% (10)	0.001
other	1.2% (6)	2.6% (13)	12% (60)	15% (78)	<0.001

* P-value from chi-squared test of homogeneity.

The percentages of children with symptoms on at least one treatment occasion are shown.

## Discussion

The results of this trial are consistent with those of a previous trial of seasonal IPT carried out in Niakhar [14] which showed a high level of protection against clinical attacks of malaria in children who received three courses of treatment with SP and 1AS given during the malaria transmission season. In the current study, four treatment regimens were investigated including the one (SP+1AS) used in the previous trial. The SP+3AQ regimen proved to be the most effective as judged by the incidence of clinical episodes of malaria and by the prevalence of parasitaemia at the end of the malaria transmission season. No placebo group was included in the current study for ethical reasons so it is not possible to calculate the efficacy of the SP+3AQ regimen directly. However, as it proved to be twice as effective as the SP+1AS regimen used in the previous study, which gave 86% protection against clinical attacks of malaria, it could be expected to have provided 93% protection. It has been questioned whether seasonal IPT would provide significant added benefit to that provided by ITNs. Overall, usage of intact untreated nets or ITNs was relatively low in the study community (18%); use of an intact net or an ITN was associated with a significantly reduced risk of a clinical attack of malaria. Analysis of the interaction between net use and seasonal IPT provided no evidence that IPT effectiveness differed between users and non-users of bednets but the study was not designed to assess this interaction and this requires further study.

The enhanced performance of the SP+3AQ regimen over those containing artesunate is likely to be due to the fact that both SP and amodiaquine have relatively long half-lives and that the protective effect of this regimen was achieved, at least in part, through chemoprophylaxis. This view is supported by the results of a trial of seasonal IPT conducted in Ghana which showed that monthly drug administration with SP+3AS was almost twice as effective as treatment given every two months (Kweku *et al.* unpublished). It seems probable that long-acting drugs will be needed for maximal effectiveness of seasonal IPT, and inclusion of artemisins in drug regimens for IPT is probably not useful due to their short action. At present SP can be used in Senegal but in other areas where there are higher levels of resistance to SP and amodiaquine this combination may be less effective for seasonal IPT. If long acting drugs are required for seasonal IPT, as seems to be the case, there are only a few possible alternatives-currently mefloquine or pyronaridine.

It is important that the need for a long-acting drug to replace SP for prophylaxis in pregnant women and perhaps in infants and young children, is born in mind by those involved in the development of new anti-malarial drugs such as the Medicines for Malaria Venture. In this study, the most effective regimen involved a three-day course of treatment and it is uncertain how well this would be followed outside the situation of a clinical trial. New prophylactic drugs should ideally be effective when given as a single treatment.

No serious adverse event attributable to study medication was observed. Minor adverse events such as vomiting, headache and subjective fever were significantly more frequent in children who received amodiaquine-containing preparations than in those who received artesunate and SP. However, overall the interventions were well tolerated. What was recorded as vomiting may include some children who spat out the medicine in reaction to the bitter taste of amodiaquine. Nevertheless, adverse events should be monitored if drugs are used on a large scale for IPT. Development of a liquid paediatric formulation would facilitate administration of IPT and might reduce minor adverse events and increase compliance.

The trial used a systematic rather than random allocation, but the method used was very unlikely to have introduced selection bias. The trial was not blinded; this could be a factor in higher rates of reported adverse events for amodiaquine-containing regimens, if there was a perception among parents of participants that the yellow tablets were more likely to cause side effects.

Intermittent treatment is likely to select for resistance. In a small study conducted in Ghana [23] this was detected after a single treatment with SP but selection for resistant parasites was not observed in a study of SP IPT in infants in Mozambique [24]. In our study, few children had malaria parasitaemia at the end of the malaria transmission season but, in those who did, nearly all parasites carried molecular markers of resistance to pyrimethamine and sulphonamides; there were no major differences between groups. The significance of this selection is uncertain. These parasites are likely to represent only a very small proportion of the parasites in the study population carrying markers of resistance to pyrimethamine (the triple DHFR mutation was present in 78% of individuals before in the start of the trial). In addition, very few of the children carrying resistant parasites were gametocytaemic and likely to transmit the infection. However, if IPT was to be used over a period of several years its impact on the parasite population would need to be monitored carefully.

Drug costs will an important issue if seasonal IPT is implemented widely. Amodiaquine is much cheaper than artesunate (currently the difference in cost is about 5 fold) so the SP+3AQ regimen has a major advantage over AS containing regimens on these grounds as well as on those of efficacy.

This study has focused on the use of anti-malarial drugs given in the community to prevent malaria. An alternative approach that is now being adopted widely in some countries, such as Uganda, is home management of febrile illnesses with anti-malarials and, as demonstrated in Ethiopia [25] this approach can reduce mortality. However, in areas of seasonal transmission of malaria there may be considerable overlap between home management and intermittent preventive treatment, unless parasitological diagnosis is performed routinely, because of the frequency of fevers in young children during the malaria transmission season. For example, in an area of The Gambia adjacent to the one in which the current study was done, home treatment for fever resulted in children aged 3 months to 9 years receiving an average of four treatments with an anti-malarial during the course of the malaria transmission season [26], a similar frequency of drug administration to that employed in this trial. Drug pressure is likely to be similar with each approach.

The results of this trial are very encouraging and show that in Senegal SP+3AQ is a highly effective regimen for intermittent preventive treatment. Tolerability and acceptability need to be carefully monitored in futures studies. Regimens containing artesunate are less effective. Thus, artesunate combinations can be reserved for treatment of clinical cases of malaria in which the rapid action of artemisinins is especially beneficial. In this trial, study drugs were administered by staff supported by the research team. Effectiveness studies are now required to identify ways in which full participation of the community can be obtained to achieve a high level of coverage with this intervention.

## Supporting Information

Protocol S1 Trial Protocol
(0.26 MB DOC)

Checklist S1 CONSORT Checklist
(0.05 MB DOC)
