# Supplementary material for: A Trial of the Efficacy, Safety and Impact on Drug Resistance of Four Drug Regimens for Seasonal Intermittent Preventive Treatment for Malaria in Senegalese Children
Source: PLoS One. 2008 Jan 23;3(1):e1471. doi: 10.1371/journal.pone.0001471 (PMC2198946; doi:10.1371/journal.pone.0001471)
Supplement: Protocol S1 — Trial Protocol (0.26 MB DOC) [file pone.0001471.s001.doc]

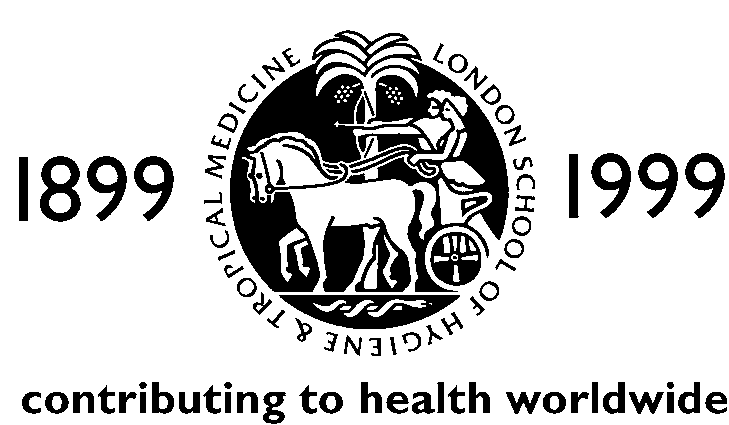


[
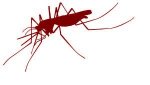
](http://www.lshtm.ac.uk/gmp/index.html)

**Gates Malaria Partnership**

**A randomised trial to compare the efficacy, safety and impact on drug resistance of four potential drugs regimens for seasonal intermittent preventive treatment in children (sIPT) in Niakhar, rural Senegal**

**LONDON – MARCH 2004**


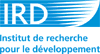


**(Confidential)**

**Correspondence to:** Dr Badara Cissé Room M/LG17 – LSHTM - 50, Bedford Square

London WC1B3DP - Email: [badara.cisse@lshtm.ac.uk](mailto:badara.cisse@lshtm.ac.uk)

**Receiving institution:** Institut de Recherche pour le Développement (IRD) Route des Pères Maristes, BP 1386, Dakar – Hann, Sénégal Telephone : +221 849 35 35- Fax : +221 832 43 07

**A randomised trial to compare the efficacy, safety and impact on drug resistance of four potential drugs regimens for sIPT in Niakhar, rural Senegal**

**Principal investigators:**

- Badara Cissé^1, 2, 3^

- Cheikh Sokhna^2^

**Co-investigators:**

- Prof. Brian Greenwood^1^, Prof. Geoffrey Targett^1^, Neal Alexander^1^, Jo Lines^1^, Colin Sutherland^1^, Rachel Hallett^1^
- Jean Francois Trape^2^, El Hadj Ba^2^
- Denis Boulanger^3^, François Simondon^3^, Franck Remoue^3^, Jacqueline Milet^3^
- Prof. Omar Gaye^4^, Prof. Oumar Faye^4^
- Adama Marra^5^

**Affiliations:**

- ^1.^London School of Hygiene and Tropical Medicine
- ^2.^Institut de Recherche pour le Développement, UR 077, Dakar Sénégal
- ^3^ Institut de Recherche pour le Développement, UR 024 Dakar & Montpellier France
- ^4^ Université Cheikh Anta Diop de Dakar (UCAD), Sénégal
- ^5.^Institut de Recherche pour le Développement, US009, Dakar Sénégal

**Sponsor:**

- IPTi Consortium – BMGF

#### Co- Sponsor:

- Impact Malaria, Sanofi-Synthelabo, 82 Ave Raspail 94255 Gentilly Cedex France

**Data Safety Monitoring Board:**

- Dr Pape Amadou Diack, clinician, coordinator of the NMCP, MoH, Dakar, chair
- Dr Khalifa Bojang, clinician MRC Fajara, member
- Dr Amadou Mbaye, clinical epidemiologist MRC Farafeni, member

#### Clinical monitor

- Prof. Masserigne Soumaré, Infectious diseases specialist, UCAD, Sénégal

**Trial Duration:**

- July 2004 to January 2005

**Total budget:**

- The approximate budget of the proposal is US$ 199 355,91

**STUDY OUTLINE**

**Rationale**

- In most of the Soudano-Sahelian zone, the burden of malaria is primarily among children between one and four years of age
- In a trial conducted in Niakhar, rural Senegal, in 2002, seasonal intermittent preventive treatment in children was found to have a marked impact on the incidence of clinical malaria (86% reduction of first clinical episode with more than 3000 parasites per micro litre)
- Results of a study implemented in the same cohort in 2003 showed the no rebound effect
- However, preliminary analysis suggests that although the children who received sIPT had a substantially lower prevalence of parasitaemia at the end of the transmission season than the control children there was a selection of parasites with molecular markers that are linked to resistance to pyrimethamine in the children who had received the intervention (three doses of SP despite the concurrent administration of one dose of artesunate.
- Because of the potential of a SP/one dose of artesunate regimen to select for pyrimethamine resistance, we have decided to explore alternative regimens for sIPT.

**Aim:**

- To improve control of malaria in the Soudano-Sahelian zone and to reduce child morbidity, by determining the best drug combination for seasonal intermittent preventive treatment in children (sIPT).

**General Objective:**

- To assess to what extent using different drug regimens can prevent morbidity from malaria in rural Senegal.

**Specific Objective:**

- To compare the efficacy, safety and effect on drug resistance of artesunate plus Sulfadoxine Pyrimethamine (SP), artesunate plus amodiaquine and Lapdap alone on malaria morbidity when used for sIPT.

**Study site:**

- Niakhar, rural Senegal

**Trial Design**:

- An open-label randomised trial using the following regimens:

- Artesunate (one day) plus SP

- Artesunate (three days) plus SP

- Artesunate (three days) plus Amodiaquine (three days)

- Lapdap (three days)

Given at monthly intervals on three occasions

**Sample size:**

- 1680 children aged 1 to 6 years (420 for each of the four arms)

**Follow-up:**

- From July 2004 to January 2005, participants will be visited at least once a week by 10 field workers
- Mothers or guardians will be asked about any episode of illness during the past seven days
- Children who have fever or any other symptoms potentially related to malaria will be follow up for at least the three following days
- Clinical attacks of malaria will be documented and treated without charge in Niakhar’s health centres by three qualified nurses
- *dhfr*, *dhps* and *pfcrt* mutations will be monitored in parasites obtained during two cross-sectional survey before and after the interventions
- This study will identify the best suitable combination for sIPT taking into account the risk of selection of drug resistant parasites

**Ethical considerations:**

- Approval will be sought from the Ethics Review Board of the Senegalese Government and from the Ethics Committee of the London School of Hygiene and Tropical Medicine.
- Informed consent will be obtained from the parents or guardians before any child is included in the study.

**Short Title:**

- Efficacy, safety and impact on drug resistance of four sIPT regimens in rural Senegal.

**Contents**

Pages

**I. Aim** 7

**II. General objectives** 7

**III. Specific Objectives** 7

# IV. Background / Rationale 7

**1. Aspects of malaria control in Sub-Saharan Africa** 7

**2. Intermittent preventive treatment (IPT)** 8

## 2.1 Definition of IPT 8

2.2 IPT in infants (IPTi) 8

2.3 IPT in children (IPTc or sIPT) 8

# V Hypotheses 12

# VI description of study 12

# 1. Study population 12

1.1. The environment 12

1.2. Demographic and epidemiological surveillance 12

1.3. Malaria transmission and mortality 12

1.4. Heath care systems in Niakhar 13

**2. Inclusion and Exclusion criteria** 13

2.1. Inclusion criteria 13

2.2. Exclusion criteria 13

**3. Study design**  13

**4. Sample size calculations** 14

**5. Enrolment procedures and follow-up** 14

**6. Randomisation and drug distribution** 14

**7. Patient assessment procedures & clinical management** 15

**8. Assessment of resistances to anti malarials** 15

**9. Assessment of adverse events** 16

**10. Participants withdrawal** 16

**11. Monitoring** 16

**12. Recruiting and training staff** 16

**13. Data management** 17

14.1. Design and organisation of the trial database 18

14.2. Data processing 18

14.3. Data analysis 18

**14. Acceptability and safety** 20

# 15. Ethical considerations 20

# 16. Information for parents and guardians 21

# 17. Timing 21

**18. Administrative matters** 21

**19. Budget** 22

# VII Implications and contributions to the Gates Programme’s objectives 23

#### Appendixes

1. Information & consent forms
2. Screening & inclusion questionnaire
3. Morbidity questionnaire
4. Clinical malaria form
5. Adverse reactions questionnaire
6. Microscopy results form
7. Consultation ticket

**References**

# I. Aim

To improve control of malaria.

# II. General objective

To assess to what extent sIPT using these different drug regimens can prevent morbidity from malaria in rural Senegal.

# III. Specific Objectives

To compare the efficacy, safety and effect on drug resistance of artesunate (one day) plus SP, artesunate (three days) plus SP, artesunate (three days) plus amodiaquine and Lapdap when used for seasonal IPT in children.

# IV. Background/Rationale

**1**. **Malaria control in Sub-Saharan Africa**

In Sub-Saharan Africa, malaria remains the most common cause of morbidity and mortality. Mortality is estimated to cause over one million deaths a year. The vast majority of this loss of life occurs among young children, especially in remote rural areas of Africa (WHO 2000).

Although tremendous advances have been made in controlling the disease during the last two decades, malaria remains the major killer in sub-Saharan Africa. Community based trials with insecticide-treated bednets (ITN) , which started in the late 1980s in the Gambia and elsewhere, achieved a 40 to 60% short term-reduction in malaria mortality in children less than 5 years in areas with high coverage (Alonso, Lindsay et al. 1993; Greenwood and Pickering 1993). However, achieving high levels of coverage with ITN has proved difficult and other control strategies are needed.

Anti malarial chemoprophylaxis for the whole paediatric population has been shown to reduce malaria morbidity and all cause mortality but this strategy is not generally encouraged due to the threat of resistance and difficulties in implementation. Chemoprophylaxis can also result in loss of acquired immunity or a delay in the development of clinical immunity leading to a period of increased risk of clinical malaria on cessation of the intervention. Any new chemotherapeutic strategy should have the potential to prevent malaria infection without compromising the development of malaria immunity or encouraging drug resistance. Intermittent preventive treatment offers a potential way of achieving these objectives. Use of drugs in combination for IPT may help to prevent selection for resistance.

**2. Intermittent preventive treatment**

2.1. Definition

IPT can be defined as drug administration, in curative doses, given at specific opportunities (for example ante-natal visits or routine vaccinations), with the objective of preventing malaria. Drugs are provided irrespective of the presence of parasites or symptoms. Drugs used for IPT must be very safe as well as effective.

2.2 Intermittent preventive treatment in infants (IPTi)

The concept of IPT was initially developed for the control of malaria in pregnancy and the administration of SP on two occasions during the second and third trimesters of pregnancy is now recommended practice in areas of medium or high malaria risk (Parise, Ayisi et al. 1998; Shulman, Dorman et al. 1999). This approach was subsequently adapted to the prevention of malaria in Tanzanian infants (Schellenberg, Menendez et al. 2001) . Administration of SP on three occasions during the first year of life at the time of EPI vaccination was found highly effective. The rate of clinical malaria (events per person-year at risk) was 0.15 in the intervention arm versus 0.36 in the placebo group, giving a protective efficacy of 59% (95%CI 41-72). In 1999, Ib Bygbjerg and colleagues studied two hundred and ninety-one infants aged 12-16 weeks who attended Maternal and Child health clinics in a Tanzanian district where malaria is holoendemic (Ib C. Bygberg et al 2002). For a period of six months, these infants were given amodiaquine or placebo every 60 days. The authors showed that the protective efficacy of intermittent amodiaquine treatment in preventing malaria fevers was 64.7% (95% C.I. 42.4 to 77.2) p<0.001. Further studies of IPTi are underway in Kenya, Ghana, Mozambique, Mali and Gabon.

2.3 Intermittent preventive treatment in children (sIPT or IPTc)

The two studies conducted in Tanzania, where malaria transmission is intense and perennial have shown that IPT during infancy has the potential to become a major tool for malaria control in highly endemic parts of Africa. However, in many parts of Africa, especially those where transmission is seasonal, the main burden of malaria is in older children who would not be protected by an IPTi scheme which is limited to infants only. Except for impregnated bednets, there are no other efficacious tools available for preventing malaria in these children. Thus, we have explored the feasibility of using the IPT approach to prevent malaria in older children.

With support from the Gates Malaria Partnership, a study was undertaken in 2002 in children in Niakhar, a typical Soudano-Sahelian area of rural Senegal where malaria transmission is seasonal and intense. A single dose of SP plus one day of artesunate were given to 600 children in September, October and November, whilst 600 other children received a placebo. The incidence of clinical malaria was reduced by 86% in children who received the intervention.

Cross-sectional surveys were undertaken before and after the administration of sIPT. The prevalence of *Plasmodium falciparum* parasitaemia at the end of the malaria transmission season in children who had received the drug combination was 69% less than in the control children (11%% versus 35%). However, molecular studies showed that there had been a selection of parasites with *dhfr* mutations associated with pyrimethamine resistance in the children who had received SP plus artesunate but not in the control group. Although there was no overall increase in the number of resistant parasites in the children who had received sIPT, because the prevalence of infection was markedly reduced in these children, this finding is a matter of concern.

A single dose of artesunate has not been effective in preventing selection for parasites carrying genetic markers of SP resistance. Although the intervention reduces the absolute number of parasites with resistant genes to SP because of the lower carriage of parasites induced, there is significant selection for resistant genotypes in the IPT group. 95% of parasites in IPT recipients carried triple mutations in *pfdhfr* compared to 75% of parasites in the placebo recipients. (figure 1).

Figure 1: Prevalence of triple mutation before and after the intervention according to the randomisation groups (Cisse B, Ba EH. & Hallett R.; unpublished data).

Therefore, the other basis of this study is to investigate if other drug regimens might be equally effective in preventing malaria but more effective in preventing the emergence of resistant parasites. Thus, we now propose to compare the efficacy, safety and impact on drug resistance of four potential drugs regimens for sIPT. The drug combinations that we have selected for comparison are:

► Artesunate (one dose) + Sulfadoxine-Pyrimethamine

SP combinations with artesunate are now of limited use in East Africa where there is widespread resistance to SP but they may still being effective in West Africa where resistance to SP is less. Thus, we chose SP + one dose of artesunate as the combination of choice for the 2002 study in Niakhar in concordance with the WHO recommendation affirming that this is a suitable combination for IPT in infants (WHO 2001). A single dose of artesunate was used because this makes compliance easier and because there is little evidence that three doses are much more effective than one dose in eliminating gametocytes. For recall, with one dose of artesunate added to SP and given in September, October and November 2002, we reached a 86% reduction of clinical malaria incidence.

►. Artesunate (three doses) + Sulphadoxine-Pyrimethamine

SP combined with artesunate given for three days will be studied in order to determine if the administration of additional doses of artesunate will eliminate any potentially SP resistant parasites and thus prevent selection during the course of the sIPT administration.

►. Artesunate + amodiaquine

The efficacy and safety of this combination for the treatment of malaria has already been documented (Brasseur, Guiguemde et al. 1999) . Studies undertaken in East and West Africa have shown that the combination of artesunate and amodiaquine is an effective treatment and may be more effective at preventing late parasitological failure than the combination of SP and artesunate when some SP resistance is present (Dorsey, Njama et al. 2002). Thus, one might think that artesunate plus amodiaquine might be more effective than SP + one dose of artesunate in preventing selection of resistance during the course of sIPT.

According to a survey conducted in 1996 in Niakhar, Senegal , 16% of parasites were resistant to amodiaquine (Sokhna, Molez et al.).

►. Lapdap

Lapdap combines two anti folates (chlorproguanil and dapsone) which both have a short half-life. This should reduce the chance of selection of resistant parasites, especially when given in combination with artesunate. Lapdap is effective against parasites that are resistant to SP, and has been shown to be safe and effective when used to treat clinical malaria in African children.

There is less information on the combination of Lapdap with artesunate. Laboratory studies have shown no adverse interactions and a trial of the combination is currently under way in Malawi. Further studies of co-administration of Lapdap with artesunate will start in a number of African countries in 2004. A fixed combination is being developed by a GSK/MMV/WHO product development team. In this efficacy trial, Lapdap will is used alone as there are yet limited safety data on the Lapdap/artesunate combination.

# V. Hypothesis

Three doses of artesunate combined with SP or amodiaquine, and Lapdap used alone will be equally as effective at preventing malaria in young Senegalese children when used for sIPT as a regimen of SP combined with one dose of artesunate and less likely to select for resistant parasites

Two major endpoints will be considered in this study (i) the incidence of clinical malaria in children receiving one of these four interventions, (ii) the prevalence of parasites carrying genotypes associated with resistance following sIPT at the end of the transmission season.

# VI Study settings

# Study population

1.1. The environment: The study will be carried out in Niakhar (Garenne M 1986; Garenne M 1990) (N 14°28’ - W 16°24’), a rural area of Central Senegal, which has appropriate conditions for such investigations. These resources are inherited from two randomised controlled trials (Samb, Aaby et al. 1993; Simondon, Yam et al. 1996) performed between 1987 and 1995. Niakhar comprises a set of 30 villages under demographic surveillance since 1983. Situated 115 km to the east of Dakar, the population comprises approximately 31 000 people living in 30 villages divided into hamlets with a total of 1 800 compounds. On average there are fourteen persons living in each compound. The site (230 square kilometres) is one of dry savannah with approximately 400-mm of rain recorded from June to September. This study area is typical of much of sub-Saharan Africa, as malaria is unstable, with transmission occurring mainly from September to November.

1.2. Demographic and epidemiological surveillance: The French Research for Development Institute (IRD web site www.ird.sn) has undertaken longitudinal follow-up of the population in Niakhar since 1983. From 1983 to 1986, field workers performed annual surveillance, during which demographic events and other epidemiological information were recorded. In 1987, in order to meet the needs of a trial of high titre measles vaccines, the system was changed to weekly home-visit of 1800 compounds.. Two supervisors controlled the quality of work done by twelve field workers. In the period 1997 – 2000, for financial reasons, this surveillance team was reduced to six field workers. Routine collections of data are now done quarterly

1.3. Malaria transmission and mortality in Niakhar: The level of malaria endemicity in Niakhar has been assessed by Vincent Robert who used three cross sectional studies conducted in February, June and November 1995 (Robert, Dieng et al. 1998). The maximal level of parasitaemia was observed in November, corresponding to the seasonal transmission peak which is also the end of rainy season. Among those 0-9 years of age, the asexual parasite rates of *Plasmodium falciparum* were 36% in February (mid-dry season), 33% in June (start of transmission season) and 79% in November (end of wet season).

In that study, entomological data showed transmission rates of ten infective bites per person per year, mainly acquired during the rainy season. Most severe cases of malaria are observed in November, particularly among children less than five years old. The latest assessments of resistance for chloroquine, amodiaquine and SP have shown respectively 44%, 16% and 7% of resistant parasites (Sokhna, Molez et al.). Surprisingly, chloroquine is still used as first-line treatment in most government facilities despite official instructions of using SP plus amodiaquine as first line. In this area, malaria mortality occurs mainly in children under 5 years of age. From 1984 to 1992, it fluctuated between 2.3 and 7.4 deaths per thousand per year with an average of 4.2 per thousand. After the emergence of chloroquine resistance, it increased to 16 per thousand per year, representing the cause of one out of four deaths in the one to four years old age group (Trape et al. 1998).

1.4. Heath cares systems in Niakhar: The site has three health centres, each of them headed by a qualified nurse. Consultations are provided daily from Monday to Friday. Pre-natal and EPI activities are normally run according to national policies. These health structures receive the assistance of an IRD physician at least one day a week.

1. Inclusion and Exclusion criteria

2.1. Inclusion criteria**.**

Eligible subjects will fulfil all of the following criteria:

αAge between 1 to 6 years of age

αInformed consent obtained from parents or legal guardians

αPermanent resident of the study area without an intention to leave it in the coming six months

αNo current participation in another malaria study

2.2. Exclusion criteria

Reasons for exclusion will include any of the following:

αAny conditions causing an inability to take or retain oral medication.

αAny chronic illnesses or severe conditions such as tuberculosis, epilepsy, obvious mental retardation or failure to thrive.

αSevere congenital diseases such as heart malformations

αIntake of an anti-malarial other than chloroquine within the previous two weeks

αKnown allergy to any of the antimalarial drugs used in the trial.

1. Study design

We will use an open label randomised trial comparing the following regimens:

- Artesunate one day plus SP

- Artesunate given for three days plus SP

- Artesunate plus Amodiaquine both given for three days

- Lapdap for three days

One of these combinations will be randomly allocated to all participants and given three times in early September, early October and early November 2004.

1. Sample size calculations

The trial is powered as an ‘equivalence’ or ‘negative’ trial, with the outcome being the cumulative incidence of malaria, ie the proportion of children having at least one episode of malaria during the course of the study. AS3+SP is the comparator and is expected to have cumulative incidence of 20%, and we aim to show that this is no more than 30% in the other three arms. To allow for three comparisons — AS3+SP compared with each of the other three arms — we set the significance level to 5/3% (≈0.0167%), one-sided. With 80% power, we need 336 children per arm (Jones, Jarvis et al. 1996). We expect no more than 20% to be lost to follow-up, which raises this to 420 per arm, or 1680 in total.

For the secondary drug resistance endpoint, this implies 336×0.2=67 cases on AS3+SP. To be conservative, we assume the same number on the other arms (if they turn out to be less effective than AS3+SP, this will only increase the power for this secondary endpoint). Among these cases, based on our previous study, we expect the prevalence of *dhfr* mutations at codons 108, 59 and 51 to be 90% in the AS1+SP arm. The 67 cases per arm then give us 77% power to detect a difference of 20% (ie 70% prevalence) in the other arms, with a significance level of 5% (two-sided, (Fleiss 1981)).

1. Enrolment procedure and follow-up

The first step will be to hold by June 2004 several community-meeting with all the partners involved in the trial including the villagers. Then, eligible children will be included if they meet the inclusion criteria and if their parents or guardians provide informed consent. The explanation provided to parents will be given in local languages, emphasising the potential public health impact for malaria control of the study (*appendix 1*). When approval is obtained after a medical examination, a study number will be allocated immediately (*appendix 2*). In addition, an ID card will be given to all participants to facilitate identification at every contact, at home or in the health centres. At the time of each drug administration, a clinical examination and an interview of parents will be performed. Children will pass through four teams respectively for identification, clinical examination, blood sampling and anti malarial drug administrations. Those who are febrile at a time of intervention will be tested for malaria and given two days treatment with Coartem® . Haemoglobin levels and drugs resistance markers will be assessed during two cross-sectional surveys conducted at the time of the first drug administration (September 04), and one month after the third and last drug administration (December 04).

1. Randomisation and drug distribution

Individual randomisation is proposed and will be done approximately three weeks before the first sIPT administration planned for early September 2004. Children will be given active drugs according to their allocation group. The investigators who do the randomisation will also separate the regimens into jars, and make a note of the code. They will therefore not be blind, but all field staff and lab staff involved directly in making observation on study subjects or their samples will be blinded to study group.

Individual notice will be given to all participants approximately one week before any intervention. Drugs will be given at the health centres.. For young children, tablets will be crushed, mixed with water and administered. All participants will be under observation for 15 minutes after drugs are taken. In case of vomiting, the drug will be re-administrated.

1. Patient assessment procedures & clinical management

Participants will be surveyed during a period of four months, September to December, with regular home visits. Morbidity screening will be carried out weekly since less frequent surveillance has been shown to underestimate the number of cases substantially. All visits will be documented using a standard form (*appendix 3*). Two days treatment with Coartem®  will be given to cases of clinical malaria defined as children who are slide positive and who have a history of fever and or vomiting within the past twenty-four hours. (Rogier, Ly et al. 1999).

A consultation ticket (appendix 7) will also be handed to the child’s parent for free consultation at the nearest health centre where physical and parasitological examinations are immediately done. Children who have been treated for malaria will be revisited at least once a day until day 3 in order to see if the treatment is effective and the child has recovered well. If parasites are still present at day 3 treatment will be pursued using Quinine after confirmation that the previous treatment had been well taken and not vomited.

For the 28 days following any confirmed malaria attack, a child will not be considered at risk for determination of malaria incidence rates.

Because follow-up will be based on passive surveillance, it is intended to organize two cross sectional surveys (September and December). Asexual forms of parasites, gametocytes and packed-cells volume will be measured for every child.

An experienced supervisor employed by the IRD will control the performance of the field-workers by randomly re-visiting in the afternoon participants seen in the morning.

Treatment will be provided for free at the three main health centres in the study area.

1. Assessment of resistances to antimalarials

At the time of the cross-sectional surveys filter paper blood samples will be collected and stored at 4 ^o^ C prior to analysis of molecular markers for resistance. For those children parasite-positive by microscopy, DNA will be extracted from the filter paper samples using standard techniques and amplified using PCR (Djimde, Doumbo et al. 2001). The reaction products will be probed for defined mutations in the dihydofolate reductase (*dhfr*), dihydroptypteroate (*dhps*) (Peterson, Di Santi et al. 1991) and *pfcrt* genes of *Plasmodium falciparum* using SSOP (Alloueche, Silveira et al. 2000). The prevalence of *dhfr* and *dhps* mutations associated with resistance to pyrimethamine will be compared between pre and post sIPT treatment samples in children who receive SP plus artesunate or Lapdap. Children who receive amodiaquine plus artesunate will act as controls. Similarly changes in the prevalence of *pfcrt* mutations before and after treatment will be measured in children who receive sIPT with amodiaquine plus artesunate whilst samples from children who receive SP or Lapdap will act as controls

1. Assessments of adverse events

Adverse events will be defined as any signs or symptoms, which first develop or increase in severity within seven days after intake. Any serious adverse events such as death or admission to hospital which occur within one week of drug administration will be thoroughly investigated and reported within 24 hours to the DSMB. To determine the prevalence of minor side effects following drug administration such as headache, fever, weakness, abdominal pain, anorexia, nausea, vomiting, diarrhoea, rash, itching or sleep disorder 100 randomly selected participants in each treatment group will be visited at home seven days after each round of drug administration and a side effects questionnaire completed. (*appendix 5*) Field staffs who complete these questionnaires will be unaware of the treatment group to which the child belongs.

1. Participants withdrawal

Parents will be at liberty to withdraw their children from the study at any time for any reason; this decision will not prejudice their children receiving malaria treatment.

Parents and legal guardians will also be informed that, on medical grounds, the principal investigator may decide to withdraw the patient. Reason(s) for this withdraw will be noted in the patient record form. In this case as well, study subjects will keep on receiving all the advantages enjoyed by continuing participants.

11. Monitoring

Clinical monitoring will be done as decided by the Consortium. Basically, a Data and Safety Monitoring Board (DSMB) will be set up with the agreement of the London School of Hygiene and Tropical Medicine (LSHTM), the Dakar University (UCAD) and the National Malaria Control Programme (PNLP). Reports of all cases of death will be given to the DSMB.

The DSMB will review the trial protocol and Standard Operating Procedures (SOPs) before the trial commences and decide on the reporting procedure that it requires.

All serious adverse events that occur within seven days of drug administration will be reported to the DSMB.

12. Recruitment and training of staff

Before commencement of the study, three nurses, two field supervisors, two drivers, seven field workers, two-biologist technicians and two data entry clerks will be recruited. A logistician and an IT manager are also needed for the study. Additional staff may be engaged when needed.

A two-day workshop will be held in early August 2004. During this meeting, the principal investigator will explain the aim of the study. Tasks will be allocated with detailed explanations of expectations for each member of the group. A formal assessment of the impact of the workshop on the participant’s understanding of the study procedures will be held. Last but not least, a strong team spirit will be built up during this retreat.

Finally a one-week pilot study will be organised including organisation of drug distribution in the field.

13. Data management

The first step of the data processing will deal with the selection of the approximately 1680 children aged from 1 to 6 years needed for the study (20% future refusal or loss to follow-up taken into account). This will be done using the demographic database of Niakhar.

13.1. Design and organisation of the trial database.

Information will be collected from two sources:

►From the Niakhar demographic database

αIdentification information of each individual: identification number, surname, first name, date of birth, gender, residency, and identification of parent

►From the sIPT trial

αDrug administration: date and place of event

αMorbidity: date of examination, symptoms

αBlood sampling: date, parasite prevalence

αAnthropometrical measurements

All this information will be structured and organised in order to have homogenous collections of data. Required files will be created accordingly. This first sequence of the work will be accomplished after detailed discussion of the questionnaires that will be used to collect information.

13.2 Data processing

Questionnaires will be completed during the active and passive surveillance conducted inside the compounds and the dispensaries.

Supervisors will check the questionnaires before they are passed to the computer desk. Supervisors will check for validity, compatibility and completeness of the returned questionnaires. Hence errors will be rapidly detected and corrected. on a daily basis, supervisors will finally go back to a random selection of compounds to verify the exactitude of data transmitted weekly by field workers.

Figure 2 shows how errors will be looked for carefully and managed before any data entry. After validation of data, transaction files will be used to update the database of the study with regards to integrity rules.

13.3 Measurements of study end-points and statistical analysis

In this study, sample size calculations are particularly done for the drug resistance end-point based on the 2002 study. Knowing that in the comparator group (AS1 + SP) the prevalence of genes with mutations markers of SP resistance increased from around 50% to 90%, the level of increase in the AS3 + SP could be looked at, with the assumption that it won’t be more than 50% to 70%.

The *dhfs*, *dhfr* and *pfcrt* genes from *Plasmodium falciparum* isolates taken on pre and post sIPT filter paper blood samples during the two cross sectional will be analysed by PCR-RFLP. Individual data will be tabulated according to the drugs regimen allocation.

The second end-point is an estimate of the effect of sIPT using the four regimens on the occurrence of the first or only clinical episode of malaria. The primary result is unadjusted protective efficacy, defined as (1-rate ratio). *Kaplan Meier Method* will be used to plot time-to-event analyses for first malaria attack after intervention, and *Log-rank* test to assess potential differences in the analyses.

Figure 2: Framework summarising the data management tasks

Back to the field

**Daily Codification &**

**Data entry**

**Weekly** checking of transaction files

**Weekly** Updating of the trial database

**Main trial database**

The database will be used to extract analysis files and to produce on a monthly basis statistical information such as:

- Basic demographic analysis of study population followed during the month (age distribution, sex ratio, geographic distribution etc…)
- Number of those followed up, lost to follow-up, died, refused and withdrawn
- Number of samples before and after the interventions
- Number of children who have been completely followed during the month
- Number of child-days a risk[[1]](#footnote-2)1
- Number of first fever, or first clinical malaria attacks
- Total number of fevers
- Total number of clinical malaria attacks
- Proportion of children free from malaria since the start of the trial[[2]](#footnote-3)2
- Proportion of children free from malaria since the last intervention
- Proportion of urine tests positive for chloroquine metabolites
- Proportion of serious adverse events after drug administration

# 14. Acceptability and safety

Tolerance and safety of the intervention will be based on analyses of observational and clinical information collected after each administration of a sIPT.

# 15. Ethical considerations

The conclusions of this study could influence malaria control policies in Senegal and in other parts of Africa. Parents and guardians will profit from the intensive surveillance activities during the study period. In addition, all malaria cases will be treated in health centres according to national policy. Thus, this study will not interfere or limit access of study participants to the national malaria prevention programme provided by the health services.

Three of the drug combinations to be used in the study, SP + one dose of artesunate, SP + three doses of artesunate and amodiaquine + artesunate have been used extensively to treat or prevent malaria in young children in Africa and their safety profiles are well known. There is less safety data for Lapdap but this drug is now licensed for the treatment of malaria in Europe and in many countries in Africa.

Informed and written consent will be obtained from parents or guardians. The study will explain the need to find an alternative to SP for seasonal IPT since this is important for future protection of their children.

Ethical approvals of the study will be sough from both:

- the Ethics review board of Research for Development Institute in France,
- the Senegalese Ethics Committee
- and the Ethics committee of the London School of Hygiene and Tropical Medicine.

#### 16. Information for parents and guardians

An explanation of the nature of the study will be provided to potential participants in *Serer* or *Wolof,* the local languages in Niakhar. An ethno linguist consultant will be used for accurate translation of the information in these dialects.

# 17. Timing

# Actually the preparatory phase will begin in June 2004 at the latest with the informed consent operations. Five months data collections are planed (interventions and malaria morbidity follow-up), starting from September 1^st^, 2004.

18. Administrative considerations

The IRD Niakhar Project (Unité de Service Niakhar) will be responsible for the administrative aspects of the study . Particular attention will be given to avoidance of any interference with other malaria studies being conducted in Niakhar. After finalisation of the protocol, no changes will be permitted without the agreement of Research Advisory Panel as well as the Niakhar Project. Any change will be formally endorsed, and attached to the original protocol.

The project has already be presented to all the senior staff of the National Malaria Programme.

19. Budget

Details of the budget are given on appendix in the budget template.

19. 1. Personnel

An experienced team is needed for this demanding study. All payments have been worked out in accordance with Senegalese salary scales.

⏵ Assistance of a computer engineer is fundamental. He will help with programming and, supervision of data entry activities. In close collaboration with the main investigator, he will be asked to produce monthly cross tabulations, which summarise the progress of work.

⏵ An assistant field physician, newly graduated from the University of Dakar will help the principal investigator to ensure medical supervision 24 hours a day and 7 days a week throughout the study.

⏵Three qualified nurses will strengthen the medical staff in existing health centres, as the trial will markedly increase the work pressures in these structures. The project activities must be complementary to health centre’s routine activities.

⏵In order to achieve good follow-up of study children 8 field workers and 2 field-supervisors are needed. This number has been carefully determined according to the average number of compounds that a field worker visits in a day.

⏵A qualified, experienced logistician will have under his responsibility all field materials

⏵ At least two drivers are needed to facilitate communication within the team. They will also ensure transportation of staff from village to village as well as between Dakar and the field.

19.2. Equipment

The following items are needed

⏵ Even if cars are for rent at the Institute which accommodate the study, it is more reliable to secure at least one personal car, as by experience we know now that such project depends a lot on logistical means. Actually four cars will be needed by at sometimes.

⏵ Seven reliable motorbikes are also needed for more efficiency of the field workers survey. They will have to visit on average forty children per day, performing required slides when necessary.

⏵ A minor equipment allowance of US$ 2805 is requested for each of the three health centres used for the trial as these structures receive only a little support from the government.

⏵A third microscope is required to help in reading on time all slides collected as an emergency

⏵A new computer is needed to speed up the data entry tasks and to allow other administrative duties inherent to such kind of trial.

⏵Finally, a small generator for the microscopes is essential to supply power cuts which are often in the study area throughout the rainy season.

19.3. Patients costs

All treatment costs and the transport costs needed for study children and their parents to reach a health centre will be reimbursed by the project for its duration. A sum of US$ 7480 is allocated for these reimbursement expenses.

19.4. Travel

Four air tickets for travelling between London and Dakar are requested; these to allow for supervision and exchange of scientists.

19.5. Institutional fees

Overheads are calculated on the basis of 10% of the total budget.

# VII Implications and contributions to the Gates Programme

Our initial study (IPT in Niakhar, Soudano-Sahelian area with seasonal transmission) has shown that this approach to malaria control has the potential to reduce malaria morbidity in children under the age of five-years substantially. However, it is uncertain which drugs regimen would be clinically highly effective whilst exerting the least drug pressure on the parasite. This new study will determine this.

It is ascertained that this project totally responds to the objectives of the Gates programme. The intervening staffs are Senegalese at 100%. Our target is to develop in the mid term a strong and operational team of prominent multi disciplinary local scientists who will then resituate their experience and training for the entire benefit of malaria control by Senegalese and for Senegalese people.

APPENDIX 1: Consent Information & Consent Form

Intermittent treatment to prevent malaria in rural Senegal

Village number |___|___| Compound number|___|___|___|

**Mother name** **Mother’s ID Number**|___|___|___|___|___|

Siblings (1=oui, 2=non)

Name ID |___|___|___|___|___| |___|

Name ID |___|___|___|___|___| |___|

Name ID |___|___|___|___|___| |___|

**Information for parents and guardians[[3]](#footnote-4)1**

Every year, when the rainy season comes, malaria re emerges in your village with its contingent of troubles for the children and sometimes even for adults. However, it is the young children who pay the heaviest tribute. Nowadays, all malaria specialists agree that the problem w will get worse unless new ways of controlling malaria are found.

We plan to run this rainy season a study that will try to assess if a new strategy found to be effective in other parts of Africa with permanent transmission of malaria can be used in our country. For that reason, we have to screen and select [2000] children aged 1 to 6years. If you give permission for your child to join the study he/she will be allocated to receive one of four different malaria drug combinations. Three of these involve malaria drugs that you will be familiar with, amodiaquine and sulphadoxine pyrimethamine given with another drug, artesunate, that is now being used increasingly in Africa and is recommended for treatment of malaria in combination with another drug by WHO.

Last year many young children in Niakhar received SP and artesunate. The drug combination was very effective in reducing malaria attacks and no serious side effects were observed. The final group of children in the new study with receive Lapdap. Lapdap is a new anti-malarial drug that is similar to SP and which has recently been licensed for use in many countries in Africa. The decision as to which drug your child receives will be made by chance. All should be protect your child from malaria but they may differ in side effects. . Children in all groups will be closely followed-up by the project with weekly home visits and free consultations at Diohine, Toucar and Ngayokheme health centres.

Since your child named X X belongs to the indicated age group, we hope that you will agree that he/she can take part in the study. In that case, in addition to drugs given, we will take a finger prick blood sample the day of drugs administration in the health centre. This specimen does not represent a health risk to your child. In the case of a possible malaria attack, the same examination will be done to make sure that the correct treatment is given. . When, malaria is detected treatment will be delivered free at Diohine, Toucar or Ngayokheme health centres for the period of the trial.

All the information collected about you and your child during the course of the study will be kept confidential, and will only be made available to the relevant authorities for the purpose of making important decisions and conclusions about the study. You are at liberty to refuse or to withdraw your child from this study at any time, and this will not prejudice him or her in regard to the offer of free health care during the season of malaria transmission. The doctor may withdraw your child from further participation if there is a medical reason why they should not continue in the study.

Any time following your child’s enrolment into the study, you are at liberty to contact Dr Badara Cisse, or his assistant, if you feel that you have not adequately been informed about the procedures, potential risks, and benefits, rights as parents of a study subject or about any other aspect of the study. The contact address for Dr Cisse is

**Consent Form**

I have been adequately informed of the purpose of this research study, the procedures, potential risks, benefits and consequences.

I understand that the drugs that my child will receive have been used before in many patients in Senegal, Gambia and many part of the world and are licensed for use in many countries and that the type of drug that my child receives will be decided by chance.

I have also understood that my child will be closely followed-up by the field workers with at least one home visit per week. In between these visits, I am at liberty to bring my child to one of three health centres for free consultation, for any worry.

I understand that I am free to withdraw the consent for my child’s further participation at any time in the course of this study, and that this will not prejudice him or her receiving free treatment in the areas’ health structures during the course of the trial.

I understand that the information obtained, as a result of my child’s participation will be treated confidential and used only by the investigator and co-investigators and the Senegalese authorities.

The investigators have answered to all my concerns.

Parent’s name: Date:

Witness:

**I have adequately informed the parents or legal guardian of the child the purpose of this study, it’s procedures, risks, benefits and consequences. I have, to the best of my ability, answered the parent’s questions regarding the study conduct and answered their concerns about the child’s participation in the study, and I will be available to continue to do so in the course of the study if need arises**

**Dr CISSE: Date:**

APPENDIX 2: Screening & inclusion questionnaire

# Date: I______I ______I______I

# Identification (Information given by the demographic and epidemiological surveillance & vaccinations card)

- Name
- ID
- Village number
- Household number
- Date of birth
- Mother’s ID
- Mother’s name
- Vaccinations dates

# Informed consent

**YES NO**

## **Inclusion criteria**

- Age between 1 months & 6 years |__| |__|
- Resident during hole malaria season transmission |__| |__|
- Has the parent or guardian given informed consent |__| |__|

## **Exclusion criteria**

- Child unable to take or to retain medications |__| |__|
- Existence of respiratory distress |__| |__|
- Existence of serious or chronic illness |__| |__|
- History of drug allergy |__| |__|
- **Is child eligible?** |__| |__|

# Anti malarial treatment received *(within the past two weeks)*

Onset treatment Duration of treatment

- Chloroquine I______I ______I______I I______I ______I______I
- Fansidar I______I ______I______I I______I ______I______I
- Quinine I______I ______I______I I______I ______I______I

- Other I______I ______I______I I______I ______I______I

# Enrolment

#### Yes or No |__| |__|

#### If yes, study number: I___I___I___I___I

**Name of the clinician or nurse who examines the child:**

APPENDIX 3: Active surveillance form

Name field worker and date of the home visit:

Name of supervisor and date Supervision:

| **ID** | **Name** | **Mother’s name** | **DOB** | **Temperature.** | **History of**  **Fever in the past 24 hours** | **History of**  **Vomiting in the past 24 hours** | **Presence in the household last night** | **Comments** |
| --- | --- | --- | --- | --- | --- | --- | --- | --- |
| 78253 | Samba Diatt | Awa Ndiaye | 26/05/99 |  |  |  |  |  |
|  |  |  |  |  |  |  |  |  |
|  |  |  |  |  |  |  |  |  |
|  |  |  |  |  |  |  |  |  |
|  |  |  |  |  |  |  |  |  |
|  |  |  |  |  |  |  |  |  |
|  |  |  |  |  |  |  |  |  |

APPENDIX 4: Passive surveillance / Diohine health centre

Name nurse/clinician and date of consultation:

Name of supervisor and date Supervision:

| **ID** | **Name** | **Mother’s Name** | **DOB** | **Date of reference** | **Date of arrival** | **Temperature** | **Diagnosis** | **Treatment** | **Comment.** |
| --- | --- | --- | --- | --- | --- | --- | --- | --- | --- |
|  |  |  |  |  |  |  |  |  |  |
|  |  |  |  |  |  |  |  |  |  |
|  |  |  |  |  |  |  |  |  |  |
|  |  |  |  |  |  |  |  |  |  |
|  |  |  |  |  |  |  |  |  |  |
|  |  |  |  |  |  |  |  |  |  |
|  |  |  |  |  |  |  |  |  |  |
|  |  |  |  |  |  |  |  |  |  |
|  |  |  |  |  |  |  |  |  |  |

### APPENDIX 5: Adverse Reactions

Village number |___|___| Compound number|___|___|___|

Mother name Mother’s ID Number|___|___|___|___|___|

Child’s name Child’s ID Number: |___|___|___|___|___|

**Notified adverse reactions** **Y** **N**

If yes,

Side effects Date onset Time onset End time Severity[[4]](#footnote-5)1

- |__________| |__________| |___________| |_____|

- |__________| |__________| |___________| |_____|

- |__________| |__________| |___________| |_____|

- |__________| |__________| |___________| |_____|

APPENDIX 6: Microscopy results

Name of the microscopist:

| **ID** | **Nom** | **Date** | Pf asexual | **Pf gametocytes** | **Other P. asexual** | **Other P. gametocytes** | **Comments** |
| --- | --- | --- | --- | --- | --- | --- | --- |
|  |  |  |  |  |  |  |  |
|  |  |  |  |  |  |  |  |
|  |  |  |  |  |  |  |  |
|  |  |  |  |  |  |  |  |
|  |  |  |  |  |  |  |  |
|  |  |  |  |  |  |  |  |
|  |  |  |  |  |  |  |  |
|  |  |  |  |  |  |  |  |
|  |  |  |  |  |  |  |  |
|  |  |  |  |  |  |  |  |

# Appendix 7: Consultation form

Reference form to be collected by the health centre nurse and given to the supervisor

Cher Docteur,

Voudriez vous consulter l’enfant nommé:

ID: Fils/fille de:

Vil/cc: Nom du CC:

Sa température à heures minutes, est de degrés

La famille signale:

Traitement d’attente reçu:

Goute épaisse faite Non faite

Name field-worker:

Signature: Date:

Date de la visite:

Nom de l’infirmier ou du médecin qui a reçu l’enfant :

Notes du superviseur :

**REFERENCES**

1. ^1^ Every child treated after confirmed malaria will lose 28 days of follow-up

   ^2^ Kaplan Meier Method will be used to plot survival curves, and Logrank test to assess potential differences in the analysis [↑](#footnote-ref-2)
2. ^2^ [↑](#footnote-ref-3)
3. ^1^ An Ethno linguist consultant will translate in the information in Serer and Wolof [↑](#footnote-ref-4)
4. ^1^ Severity: 1=Mild, 2=Moderate, 3=Severe [↑](#footnote-ref-5)
